# Supplementary material for: DBU-Net: Dual branch U-Net for tumor segmentation in breast ultrasound images
Source: PLoS One. 2023 Nov 6;18(11):e0293615. doi: 10.1371/journal.pone.0293615 (PMC10627442; doi:10.1371/journal.pone.0293615)
Supplement: Attachment — Submitted filename: review.docx [file pone.0293615.s001.docx]

The authors propose a paper on “DBU-Net: Dual Branch U-Net for Breast Ultrasound Image Segmentation". I believe that the manuscript is well organized and explained. Following comments can improve its quality.

1. The authors should follow this order for your revised abstract: (1) Novelty (2) Methods (3) Results (the most noticeable numbers) (4) conclusion. Results are of high significance, and they need to provide the most important outcomes here. Please revise the abstract accordingly.
2. Results ratio miss and also cross validation
3. Introduction section is weak. I would recommend to split it in problem, objectives, literature, and contribution.
4. Is this model applicable for color model if yes then why not use it
5. Please, report the recent medical image analysis challenges. Moreover, the paper can be strengthened with technical and terminology innovations by may be considering the below recent manuscripts:
   1. Rauf, Zunaira, Anabia Sohail, Saddam Hussain Khan, Asifullah Khan, Jeonghwan Gwak, and Muhammad Maqbool. "Attention-guided multi-scale deep object detection framework for lymphocyte analysis in IHC histological images." Microscopy (2022).
   2. Zahoor, M.M.; Qureshi, S.A.; Bibi, S.; Khan, S.H.; Khan, A.; Ghafoor, U.; Bhutta, M.R. A New Deep Hybrid Boosted and Ensemble Learning-Based Brain Tumor Analysis Using MRI. Sensors 2022, 22, 2726. <https://doi.org/10.3390/s22072726>
   3. Khan, Saddam Hussain, Javed Iqbal, Syed Agha Hassnain, Muhammad Owais, Samih M. Mostafa, Myriam Hadjouni, and Amena Mahmoud. "Covid-19 detection and analysis from lung ct images using novel channel boosted cnns." Expert Systems with Applications 229 (2023): 120477.
   4. Khan, Saddam Hussain, Najmus Saher Shah, Rabia Nuzhat, Abdul Majid, Hani Alquhayz, and Asifullah Khan. "Malaria parasite classification framework using a novel channel squeezed and boosted CNN." Microscopy 71, no. 5 (2022): 271-282.
6. Are there any methodology limitations and/or challenges required to be addressed? All these should be mentioned at the end of the Conclusions section.
7. Why you select relu function as activation function why not Sigmoid, Tanh, ReLU, Leaky ReLU, PReLU, ELU, and SELU. Do compression in form of table with other activation function.
8. In data set you mentioned three classes how you make classes  normal, benign, and malignant.
9. Uneven dataset it will give False positive
10. Fig 5 and 7 are not visible please update them with readable legends.
11. What is the author’s contribution? Please include paragraph with comparison with recent literature.
12. ROC and confusion matrix
13. Contribution section must be cited with recent paper what novel aspect authors achieved.
14. Table 7 must be elaborated how these different model lacks what’s author’s criteria?
15. All equations must be cited if are not authors work.
16. Future direction are too generic.
