# Supplementary material for: DBU-Net: Dual branch U-Net for tumor segmentation in breast ultrasound images
Source: PLoS One. 2023 Nov 6;18(11):e0293615. doi: 10.1371/journal.pone.0293615 (PMC10627442; doi:10.1371/journal.pone.0293615)
Supplement: Attachment — Submitted filename: Response sheet_PONE-D-23-21799.docx [file pone.0293615.s002.docx]

**Manuscript ID: PONE-D-23-21799**

We are very grateful to the Editor for considering our manuscript for review and we are very thankful to the reviewers for providing us with valuable suggestions and giving feedback about our manuscript. We have made the required changes to address the editor’s and reviewers’ comments. Please note that the changes are highlighted in the revised manuscript and herein as blue for Reviewer #1 and green for Reviewer #2 for the convenience of the reviewers. Replies to each of the reviewers’ comments are also listed below.

—---------------------------------------------------------------------------------------------------------

**Additional Editor Comments:**

Please implement the proposed technique on UDIAT and other breast cancer ultrasound images to enhance the dataset's robustness and make it available to researchers, thereby accelerating research endeavors.

**Response:** Thank you for your suggestions. Please note that we have evaluated our method on the UDIAT dataset also, as suggested by the reviewers. Please refer to **Table 8** and **Figure 13** for the detailed results. Also, UDIAT is not a publicly accessible dataset. However, this dataset can be found/accessed by requesting the developers through the process mentioned on the website (<http://www2.docm.mmu.ac.uk/STAFF/M.Yap/dataset.php>).

—--------------------------------------------------------------------------------------------------------

**Reviewer’s Comments:**

**Reviewer #1:**

**Comment #1:** The abstract lacks clarity regarding the novelty of the proposed approach, omits details on validation techniques and empirical results, and requires improvement in grammatical and technical English to enhance readability.

**Response:** We are grateful to the reviewer for his/her valuable feedback on our manuscript. We have tried our best to improve the quality of the manuscript based on the very useful suggestions provided by the reviewers. We have improvised the **Abstract** with the clear information about our proposed method in the revised manuscript. We have also corrected the technical English to improvise on readability.

**Comment #2:** The quality of figures needs to be enhanced for better readability and comprehension.

**Response:** Thank you very much for pointing out this shortcoming, Kindly note that based on your observation we have updated the figures with enhanced quality.

**Comment #3:** The sentence structure needs improvement to avoid repetition of similar words, particularly when referring to table names.

**Response:** Thank you for letting us know this shortcoming. We have improved the sentence structures throughout the manuscript to avoid repetition of similar words.

**Comment #4:** The novelty of the proposed technique based on existing deep UNet CNN should be elaborated in detail, providing a rationale for the approach and discussing the potential impact of the techniques.

**Response:** Thank you for your useful suggestions. Kindly note that we have revised the manuscript by mentioning the novelty of the proposed work based on the research gaps found in existing methods.

**Comment #5:** To compute the results, it is suggested to use UDIAT in conjunction with the BUS dataset. Additionally, making the datasets public would facilitate further research activities.

**Response:** Thank you for your useful suggestion for adding one more dataset to evaluate our method. Please note that we have evaluated our method on the UDIAT dataset also, as suggested. Please refer to **Table 8** and **Figure 13** for the detailed results.

We would also like to mention here that BUSI is a publicly available dataset (downloadable link is given in the paper). But UDIAT is not a publicly accessible dataset. However, this dataset can be found/accessed by requesting the developers through the process mentioned on the website (<http://www2.docm.mmu.ac.uk/STAFF/M.Yap/dataset.php>).

**Comment #6:** A detailed ablation study, including results and computational complexity, should be provided to better understand the effectiveness and efficiency of the proposed method.

**Response:** Thank you for your constructive comments and suggestions. Please note that we have described the detailed results, comparative studies, and a statistical test. Please refer to **Tables 3-6.** We have also provided a detailed analysis of our results in the subsection namely **Analysis of the Segmentation Results (Page 14)**.

**Comment #7:** Enhancing the novelty of the paper can be achieved by considering recent medical challenges and incorporating technical innovations and concepts in deep segmentation CNN may be considering the below manuscripts:

a. Zafar, Muhammad Mohsin, Zunaira Rauf, Anabia Sohail, Abdul Rehman Khan, Muhammad Obaidullah, Saddam Hussain Khan, Yeon Soo Lee, and Asifullah Khan. "Detection of tumour infiltrating lymphocytes in CD3 and CD8 stained histopathological images using a two-phase deep CNN." Photodiagnosis and Photodynamic Therapy 37 (2022): 102676..

b. Khan, Saddam Hussain, Asifullah Khan, Yeon Soo Lee, Mehdi Hassan, and Woong Kyo Jeong. "Segmentation of shoulder muscle MRI using a new region and edge based deep auto-encoder." Multimedia Tools and Applications 82, no. 10 (2023): 14963-14984.

c. Zahoor, Mirza Mumtaz, and Saddam Hussain Khan. "Brain tumor MRI Classification using a Novel Deep Residual and Regional CNN." arXiv preprint arXiv:2211.16571 (2022).

d. Khan, Saddam Hussain. "COVID-19 Detection and Analysis From Lung CT Images using Novel Channel Boosted CNNs." arXiv preprint arXiv:2209.10963 (2022).

e. Khan, Saddam Hussain, Najmus Saher Shah, Rabia Nuzhat, Abdul Majid, Hani Alquhayz, and Asifullah Khan. "Malaria parasite classification framework using a novel channel squeezed and boosted CNN." Microscopy 71, no. 5 (2022): 271-282.

**Response:** Thank you very much for suggesting the above mentioned research articles. Please note that we have reviewed these suggested papers and incorporated the challenges found therein in our revised manuscript.

**—---------------------------------------------------------------------------------------------------------
Reviewer #2:**

The authors propose a paper on “DBU-Net: Dual Branch U-Net for Breast Ultrasound Image Segmentation”. I believe that the manuscript is well organized and explained. Following comments can improve its quality.

**Comment #1:** The authors should follow this order for your revised abstract: (1) Novelty (2) Methods (3) Results (the most noticeable numbers) (4) conclusion. Results are of high significance, and they need to provide the most important outcomes here. Please revise the abstract accordingly.

**Response:** We are grateful to the reviewer for his/her valuable feedback on our manuscript. We have tried our best to improve the quality of the manuscript based on the very useful suggestions provided by the reviewers. We have improvised the abstract in our revised manuscript as per the suggestion provided by the reviewer.

**Comment #2:** Results ratio miss and also cross validation

**Response:** We appreciate the reviewer's concern in this. Please note that we have conducted the 5-fold cross validation experiment and the results are shown in **Table 3** and **Table 4**. In 5-fold cross validation, we split the entire dataset into equal 5 groups and the model is trained on 4 out of 5 groups and the remaining group is used for testing.

**Comment #3:** Introduction section is weak. I would recommend to split it in problem, objectives, literature, and contribution.

**Response:** Thank you for your constructive suggestions that help us to improve the paper. Please note that we have revised the Introduction section following your suggestions.

**Comment #4:** Is this model applicable for color model if yes then why not use it

**Response:** We appreciate the reviewer's concern in this. Please note that most of the previous work is done using the gray scale images for this particular dataset, and in this work, we have followed the standard literature.

**Comment #5:** Please, report the recent medical image analysis challenges. Moreover, the paper can be strengthened with technical and terminology innovations by may be considering the below recent manuscripts:

1. Rauf, Zunaira, Anabia Sohail, Saddam Hussain Khan, Asifullah Khan, Jeonghwan Gwak, and Muhammad Maqbool.”Attention-guided multi-scale deep object detection

framework for lymphocyte analysis in IHC histological images.” Microscopy (2022).

2. Zahoor, M.M.; Qureshi, S.A.; Bibi, S.; Khan, S.H.; Khan, A.; Ghafoor, U.; Bhutta, M.R. A New Deep Hybrid Boosted and Ensemble Learning-Based Brain Tumor Analysis

Using MRI. Sensors 2022, 22, 2726. https://doi.org/10.3390/s22072726

3. Khan, Saddam Hussain, Javed Iqbal, Syed Agha Hassnain, Muhammad Owais, Samih M.Mostafa, Myriam Hadjouni, and Amena Mahmoud. &quot;Covid-19 detection and analysis from lung ct images using novel channel boosted cnns.&quot; Expert Systems with Applications 229 (2023): 120477.

4. Khan, Saddam Hussain, Najmus Saher Shah, Rabia Nuzhat, Abdul Majid, Hani

Alquhayz, and Asifullah Khan. “Malaria parasite classification framework using a novel channel squeezed and boosted CNN.”Microscopy 71, no. 5 (2022): 271-282.

**Response:** Thank you so much for suggesting some important research articles. Please note that we have reviewed these suggested papers and incorporated those in our revised manuscript.

**Comment #6:** Are there any methodology limitations and/or challenges required to be addressed? All these should be mentioned at the end of the Conclusions section.

**Response:** Thank you for your very useful suggestions. Please note that we have mentioned the limitations of our method at the end of the **Analysis of the Segmentation Results section,** as suggested by the reviewer.

**Comment #7:** Why you select relu function as activation function why not Sigmoid, Tanh, ReLU, Leaky ReLU, PReLU, ELU, and SELU. Do compression in form of table with other activation function.

**Response:** Thank you for this very interesting question. In this context we would like to state that for activation functions like Tanh, Sigmoid and ELU, these belong to the category of exponential curves. Using such an activation function also yields the derivatives to be exponential, as a result of this the learning rate needs to be rapidly changed and adapted accordingly, thus the entire model is tricky to train. On the other hand activation functions like Leaky ReLU, PReLU have extra hyper-parameters/parameters which need to be manually finetuned which is not very straightforward at times. Thus, the use of ReLU based activation in addition to imparting non-linearity to the learning process also alleviates the need of any complex methodologies to address the above issues.

**Comment #8:** In data set you mentioned three classes how you make classes normal, benign, and malignant.

**Response:** We appreciate the reviewer's concern on this. Please note that the dataset is publicly accessible at <https://scholar.cu.edu.eg/?q=afahmy/pages/dataset> and in the dataset itself the class folders are already provided there; we have not made any classification.

**Comment #9:** Uneven dataset it will give False positive

**Response:** We appreciate the reviewer's concern on this. We agree that the dataset is uneven and we have taken care of this in our proposed model which can be seen in **Figure 7**. Also, we have discussed the error case analysis where we have shown the cases in which our model produces false positive results.

**Comment #10:** Fig 5 and 7 are not visible please update them with readable legends.

**Response:** We thank the reviewer for pointing this out. Please note that we have enhanced the quality of figures with readable legends.

**Comment #11:** What is the author’s contribution? Please include paragraph with comparison with recent literature.

**Response:** Thank you for your comment. Please note that the contribution of the proposed method is discussed at the end of the **Introduction** section pointwise.

**Comment #12:** ROC and confusion matrix

**Response:** We thank the reviewer for suggesting this. Please note that we have considered the evaluation metrics by following the previous segmentation works. ROC curves and confusion matrices are suited for classification tasks, where input images are assigned predefined classes, unlike image segmentation where each pixel is labeled. Image segmentation metrics like IoU, Dice coefficient measure the overlap between predicted and ground truth masks which is more suitable to evaluate a segmentation task that’s why we have used these two matrices.

**Comment #13:** Contribution section must be cited with recent paper what novel aspect authors achieved.

**Response:** Thank you for your suggestions and feedback. Please note that we have added many recent papers, and mentioned the novelty of our method keeping in mind the research gaps found in existing methods.

**Comment #14:** Table 7 must be elaborated how these different model lacks what’s author’s criteria?

**Response:** Thank you for your suggestions. Please note that we have provided a detailed analysis of results shown in **Table 7**.

**Comment #15:** All equations must be cited if are not authors work.

**Response:** We appreciate the reviewer’s concern on this. Please note that we have taken care of this.

**Comment #16:** Future direction are too generic.

**Response:** Thank you reviewer for pointing this out. Please note that we have updated the future direction portion with some specific solution in our revised manuscript.
